# Oxidative stress-related biomarkers in thyroid eye disease: evidence from bioinformatics analysis and experimental validation

**DOI:** 10.3389/fimmu.2025.1635712

**Published:** 2025-08-18

**Authors:** Yuanping Hai, Qintao Ma, Zhitao Liu, Dongxiao Li, Anqi Huang, Yan Zhu, Duan Yongbo, Cheng Song, Genfeng Yu, Sijie Fang, Lan Liu, Yi Wang, Thomas Efferth, Jie Shen

**Affiliations:** ^1^ Department of Endocrinology and Metabolism, The Eighth Affiliated Hospital of Southern Medical University (The First People’s Hospital of Shunde Foshan), Foshan, Guangdong, China; ^2^ Department of Pharmaceutical Biology, Institute of Pharmaceutical and Biomedical Sciences, Johannes Gutenberg University, Mainz, Germany; ^3^ Department of Orthopedics, People’s Hospital of Ningxiang City, Hunan University of Chinese Medicine, Changsha, China; ^4^ Department of Ophthalmopathy, The Eighth Affiliated Hospital of Southern Medical University (The First People’s Hospital of Shunde Foshan), Foshan, China; ^5^ Department of Ophthalmology, Shanghai Ninth People’s Hospital, Shanghai Jiao Tong University School of Medicine, Shanghai, China; ^6^ Department of Ophthalmology, Peking University Third Hospital, Beijing, China; ^7^ Beijing Key Laboratory of Restoration of Damaged Ocular Nerve, Peking University Third Hospital, Beijing, China

**Keywords:** ANGPTL7, artificial intelligence, biomarkers, FOS, immunohistochemistry, MCL1, oxidative stress, thyroid eye disease

## Abstract

**Background:**

Oxidative stress is a key contributor to the pathogenesis of the autoimmune condition thyroid eye disease (TED). However, its precise molecular mechanisms and reliable biomarkers remain unclear. Bioinformatics enables the identification of differentially expressed genes through transcriptomic analysis. However, distinguishing truly relevant findings from false discoveries remains challenging. Immunohistochemistry helps address this limitation by validating protein expression levels, revealing local immune responses, and linking microscopic tissue changes to clinical manifestations.

**Methods:**

Oxidative stress-related differentially expressed genes (OS-DEGs) were identified. Gene Ontology (GO) and Kyoto Encyclopedia of Genes and Genomes (KEGG) pathway analyses were performed to explore their biological functions and pathways. Machine learning methods, including LASSO regression and random forest, were used to select key diagnostic genes. Receiver operating characteristic curves assessed their diagnostic performance. A nomogram model was constructed using logistic regression based on selected oxidative stress-related core genes. Single-gene gene set enrichment analysis evaluated the diagnostic potential and functional relevance of these core genes. Expression of three key genes/proteins repeatedly highlighted in multi-omics TED studies was confirmed in 22 orbital tissues by immunohistochemistry with quantitative analysis using automated image tools minimizing operator bias.

**Results:**

Fifty-three OS-DEGs were selected. GO and KEGG enrichment analyses revealed significant involvement of OS-DEGs in cellular responses to oxidative stress, ROS metabolism, and mitochondrial dysfunction, highlighting the role of oxidative damage in TED. Five diagnostic genes (*AKT1, APEX1, FOS, MCL1*, and *ANGPTL7*) were identified through machine learning approaches (LASSO regression and random forest), demonstrating strong diagnostic potential with a combined model achieving an area under the curve (AUC) of 0.931. The nomogram model developed using the selected genes showed good predictive performance for TED risk assessment. Immunohistochemical validation confirmed significant upregulation of FOS, MCL1, and ANGPTL7 in TED *versus* controls.

**Conclusions:**

To the best of our knowledge, this study is the first to identify three oxidative stress-related genes/proteins as potential biomarkers for TED through bioinformatic analysis of multi-omics data followed by immunohistochemical validation, providing new insights into their roles in the pathogenesis of the disease. These biomarkers could aid in early screening and risk assessment for TED.

## 1 Introduction

Thyroid eye disease (TED) is a chronic autoimmune orbital disorder associated with thyroid dysfunction, characterized by inflammation, tissue remodeling, and fibrosis (1–4). Its pathogenesis involves disruption of immune tolerance, leading to infiltration of immune cells — such as macrophages and lymphocytes — into orbital tissues and the release of inflammatory mediators (5–8). As key effector cells, orbital fibroblasts (OFs) differentiate into myofibroblasts or adipocytes under inflammatory stimuli, promoting fibrosis or fat expansion and contributing to proptosis (7, 9, 10). Activated OFs also produce glycosaminoglycans and cytokines, further aggravating orbital inflammation and tissue remodeling (1, 7, 8, 11).

In recent years, oxidative stress has garnered increasing attention for its critical role in TED progression (6, 12–16). Oxidative stress refers to an imbalance between the production and elimination of reactive oxygen species (ROS), which promotes OF proliferation and induces the expression of the 72 kDa heat shock protein, further increasing ROS levels and exacerbating oxidative stress (1, 6). Oxidative stress-related molecules, including superoxide dismutase, superoxide anion, malondialdehyde, hydrogen peroxide, and glutathione reductase are significantly elevated in OFs from TED patients compared to healthy controls (6, 14). ROS are natural byproducts of cellular metabolism and play essential roles in maintaining cellular homeostasis and signaling. However, excessive ROS levels or deficiencies in antioxidant defense mechanisms lead to oxidative damage to proteins, lipids, membranes, and nucleic acids, ultimately resulting in mitochondrial dysfunction and loss of enzymatic activity (12, 13, 15, 16). Several studies have confirmed increased oxidative damage markers in blood, urine, and tear samples from TED patients (13). Blood samples from TED patients show elevated levels of hydrogen peroxide and lipid hydroperoxides along with increased activity of antioxidant enzymes such as superoxide dismutase and catalase, whereas glutathione peroxidase and glutathione reductase activities are reduced (12, 13). Furthermore, ROS promote OFs proliferation, glycosaminoglycan secretion, and the release of pro-inflammatory cytokines and various inflammatory mediators, thereby aggravating TED pathogenesis (1). Therefore, elucidating the oxidative stress mechanisms underlying TED could provide new insights into disease management and therapeutic strategies.

Redox signaling pathways play key roles in TED progression by upregulating potent inflammatory cytokines such as interleukin (IL)-1β, IL-6, and transforming growth factor (TGF)-β, thereby promoting inflammation, adipogenesis, and fibrosis (13). In addition, oxidative stress induced by smoking is one of the most significant risk factors for TED, whereas antioxidant interventions such as selenium supplementation may help mitigate disease progression (15, 16). Several *in vitro* studies demonstrated that bioactive components from traditional Chinese medicine may have therapeutic potential by suppressing oxidative stress in OFs (1, 13). Although the pathogenic role of oxidative stress in TED is widely recognized, its precise molecular mechanisms remain unclear.

Recent advances in bioinformatics and genomic technologies have enabled the identification of differentially expressed genes and signaling pathways through transcriptomic analysis (3–5, 17, 18). However, the association between oxidative stress and TED has not been systematically explored using these methods. Although bioinformatic tools are effective for discovering disease-related targets, they are limited by false positives and lack experimental confirmation. To address this, we used immunohistochemistry to validate the identified targets, providing protein-level evidence and reflecting local immune activity linked to TED pathology (5, 9). Previous immunohistochemical studies have contributed to elucidating TED pathogenesis but often depended on operator-based assessments (9, 10). In contrast, our study utilized a validated and fully automated software platform to conduct quantitative analysis, thereby enhancing the objectivity and reproducibility of the results (5, 9).

We employed transcriptomic sequencing and bioinformatics analysis to identify five core oxidative stress-related genes (*AKT1, APEX1, FOS, MCL1, and ANGPTL7*) associated with TED. Subsequently, a literature review and analysis of original data from these publications revealed that *FOS, MCL1*, and *ANGPTL7* were identified in TED-related multi-omics studies (3–5, 19–23), including single-cell RNA sequencing (21) and our previously published transcriptomic (3), bioinformatic analyses (4), and high-throughput proteomic analyses (5). Furthermore, immunohistochemical validation was performed to confirm the expression of FOS, MCL1, and ANGPTL7 at the protein level, aiming to elucidate the oxidative stress mechanisms underlying the pathogenesis of TED and to provide a theoretical basis for precision diagnosis and treatment.

## 2 Methods

### 2.1 Patients and samples

This study was conducted in accordance with the principles outlined in the Declaration of Helsinki and was approved by the Institutional Review Board of Shunde Hospital, Southern Medical University (Ethics approval No. KYLS20240815). Written informed consent was obtained from all participants prior to their inclusion in the study.

A total of 22 orbital tissue samples were collected from 22 individuals at the Department of Ophthalmology, Shunde Hospital, Southern Medical University (Foshan, China). Of these, 12 TED samples — comprising six from patients with clinically active and severe TED, and six from those with inactive and mild TED — were obtained during urgent decompression or rehabilitative orbital decompression surgeries. The remaining 10 specimens were harvested during blepharoplasty procedures from individuals without any history of autoimmune, orbital, or thyroid disorders. Immediately after surgical excision, tissues were snap-frozen in liquid nitrogen and stored at −80 °C. Prior to immunohistochemical analysis, all samples were processed using the formalin-fixed, paraffin-embedded technique.

### 2.2 Collection of public transcriptomic data and oxidative stress-related differential gene identification

We extracted a total of 458 oxidative stress-related genes (OSRGs) from the Gene Ontology (GO) knowledgebase (http://geneontology.org/). The GSE58331 dataset was obtained from the Gene Expression Omnibus (GEO) database (http://www.ncbi.nlm.nih.gov/geo), comprising 27 orbital tissue samples from TED patients and 22 samples from healthy individuals. Probe annotation files from the corresponding platforms were utilized to map probes to gene symbols. Differentially expressed genes (DEGs) in retro-orbital tissues of TED patients and healthy controls were identified using the “limma” package, with the threshold set at *p* < 0.05 and |fold change (FC)| > 0.3. The identified DEGs were then intersected with oxidative stress-related genes to obtain oxidative stress-related differential genes (OS-DEGs).

### 2.3 Enrichment analyses of OS-DEGs

We utilized the clusterProfiler package in R to perform GO and Kyoto Encyclopedia of Genes and Genomes (KEGG) pathway enrichment analyses on OS-DEGs, aiming to explore their biological functions and significance. A threshold of *p* < 0.05 was set for statistical significance. Additionally, the enrichment results were visually represented using bubble plots and bar charts.

### 2.4 Screening oxidative stress-related core genes in TED

This study employed LASSO regression and random forest (RF) machine learning algorithms to identify key oxidative stress-related genes in TED. LASSO regression, a widely used dimension reduction method, excels in high-dimensional data analysis by incorporating regularization to enhance predictive accuracy. A 10-fold cross-validation was performed using the “glmnet” package in R, optimizing the model through a tuning penalty parameter. Additionally, we applied RF, a supervised learning algorithm based on decision trees, to further refine the selection of hub genes. Feature importance was assessed using the Mean Decrease Gini Index, which quantifies the contribution of each variable to classification performance. The intersection of genes identified by both methods was considered as the oxidative stress-related core gene set. The expression levels of these core genes between groups were visualized using boxplots. Furthermore, to evaluate their diagnostic potential, we generated receiver operating characteristic (ROC) curves and used the “pROC” package in R to calculate the area under the curve (AUC), providing a quantitative measure of diagnostic efficiency.

### 2.5 Development of an oxidative stress-related nomogram for TED

We utilized the “rms” package in R to develop a nomogram model based on the selected oxidative stress-related core genes. The total score generated by the model exhibited a positive correlation with the risk of TED occurrence. The nomogram included “points”, representing the individual scores of each candidate gene, and “total points”, which reflected the cumulative gene scores. The probability of TED occurrence for an individual was estimated by mapping the total score to the corresponding risk level. To assess the predictive performance of the model, we conducted calibration curve analysis, decision curve analysis (DCA), and clinical impact evaluation, ensuring its accuracy and clinical applicability.

### 2.6 Gene set enrichment analysis for single genes

In this study, gene set enrichment analysis (GSEA) was performed to functionally annotate samples with varying expression levels of target genes. Samples were first stratified into high-expression and low-expression groups based on the median expression level of the target gene. The clusterProfiler package was utilized for the GSEA analysis, using the c5.go.Hs.symbols.gmt gene set file to identify biological processes significantly associated with the expression of the target gene. The log fold change (logFC) values between the two groups were calculated, and pathways with a p-value < 0.05 were considered significantly enriched. Finally, for each target gene, the top five significantly upregulated pathways were selected and visualized through enrichment plots, providing deeper insights into the underlying biological mechanisms.

### 2.7 Immunohistochemical procedure

Tissue sections were deparaffinized using an environmentally friendly dewaxing solution for 30 min and rehydrated in anhydrous ethanol for 15 min. Epitope retrieval was performed in 20× Tris-EDTA buffer (pH 8.0) using a microwave oven for 30 minutes. Non-specific background staining was blocked with 3% hydrogen peroxide and a protein blocking reagent for 25 and 30 min, respectively. The sections were then incubated overnight at 4 °C with the following primary antibodies and dilutions: FOS (1:300; Servicebio, Cat# GB11069), MCL1 (1:200; Servicebio, Cat# GB11696), and ANGPTL7 (1:300; Proteintech, Cat# 10396-1-AP). Signal amplification was carried out using HRP-conjugated secondary antibodies corresponding to the species of the primary antibodies, followed by incubation at room temperature for 50 min. Chromogen detection was performed using freshly prepared DAB solution. Hematoxylin was used for counterstaining, and tissue sections were dehydrated through a graded ethanol series (75%, 85%, 100%), each for 5 minutes. Finally, slides were cleared in xylene for 5 min and sealed with mounting medium.

### 2.8 Quantitative analysis of immunostaining

The immunostained slides were scanned using the Panoramic scanner (3DHISTECH Ltd., Budapest, Hungary) and subsequently analyzed with DensitoQuant software through the Panoramic Viewer platform. Positive signals were detected via automated color deconvolution, which differentiated pixel intensity levels — brown indicating positive immunoreactivity and blue serving as a negative counterstain. The proportion of positive cells was determined by dividing the number of positively stained cells by the total cell count across six separately annotated regions of orbital tissue (5, 9).

For each immunostained slide, six representative areas were selected separately from the orbital connective tissue and orbital adipose tissue (**Supplementary Figure S1**) as our previously studied (5, 9). All sections stained with the same antibody were analyzed under uniform parameter settings in the DensitoQuant software. The extent of positive staining was quantified based on the software’s built-in algorithm. The average value across the six annotated regions was calculated to represent the protein expression level for each tissue type. Consistent application of analysis parameters across all samples ensured reproducibility and allowed for fast, automated, and operator-independent quantification. Statistical analyses were conducted using the Statistical Package for the Social Sciences. Independent t-tests were employed to compare differences between two groups. A *p*-value less than 0.05 was regarded as statistically significant. Data visualization and graph generation were carried out using Prism software.

## 3 Results

### 3.1 Demographic and clinical data

Demographic and clinical data are presented in **Table 1**.

**Table 1:** Demographic and clinical data.

Indicators	Active GO (n=6)	Inactive GO (n=6)	Control (n=10)
Age (year)	54 ± 5.3	33.2 ± 6.2	35.8 ± 15.3
Gender, female *N* (%)	3 (50)	6 (100)	7 (70)
CAS (point)	3.7 ± 0.5	0.3 ± 0.5	N/A
Duration (year)	0.6 ± 0.2	5.0 ± 3.1	N/A
Orbital irradiation ratio *N* (%)	0 (0)	0 (0)	N/A
Smoker	1 (17)	0 (0)	N/A
Drinker	0 (0)	0 (0)	N/A
Severe *N* (%)	6 (100)	0 (0)	N/A
Mild *N* (%)	0 (0)	6 (100)	N/A
Steroid therapy *N* (%)	6 (100)	6 (100)	N/A

Data are shown as mean ± standard deviation. CAS, clinical activity score; N, number; N/A, not available; Duration, duration of orbital disease.

### 3.2 Identification of oxidative stress-related DEGs associated with TED

**Figure 1:**
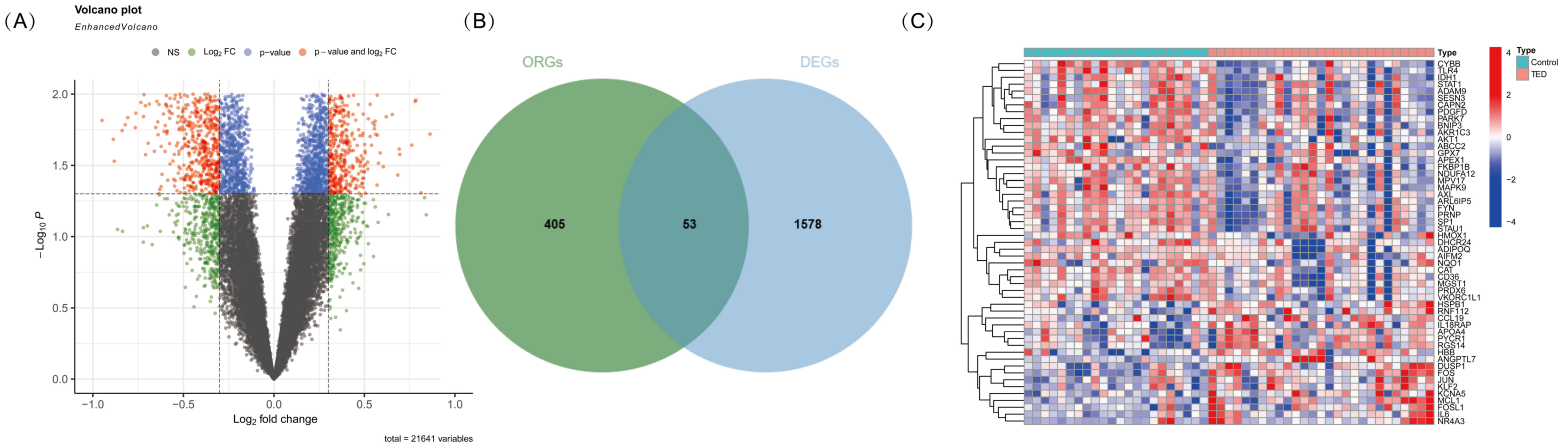
**(A)** Volcano plot illustrating differentially expressed genes (DEGs) in TED and control samples. The x-axis represents the log2 fold change, while the y-axis represents the -log10 *p*-value. Genes with significant upregulation (red). **(B)** Venn diagram showing the intersection of DEGs with oxidative stress-related genes. The overlapping region represents oxidative stress-related differentially expressed genes (DE-OSRGs). **(C)** Heatmap displaying the expression patterns of DE-OSRGs in TED and control samples. Each column represents an individual sample, while each row represents a gene. The color scale indicates relative expression levels, with red representing high expression and blue representing low expression.

To identify oxidative stress-related differentially expressed genes (DE-OSRGs) in TED, we first screened for DEGs using the threshold log2|FC| > 0.3 and *p*-value < 0.05 as our previously studied (4), identifying a total of 1,631 DEGs. The volcano plot (**Figure 1A**) presents the overall distribution of these DEGs, highlighting significantly upregulated and downregulated genes between TED and control samples. Next, we intersected the identified DEGs with a predefined set of 458 OSRGs, resulting in 53 DE-OSRGs. The Venn diagram (**Figure 1B**) illustrates this intersection process, showing the overlap between the oxidative stress-related gene set and the identified DEGs. To further explore the expression patterns of these 53 DE-OSRGs, we generated a heatmap (**Figure 1C**), which clearly differentiates TED and control groups based on gene expression profiles. This analysis helped identify potential oxidative stress-related biomarkers that may play a key role in TED pathogenesis.

### 3.3 Enrichment analysis of DE-OSRGs

**Figure 2:**
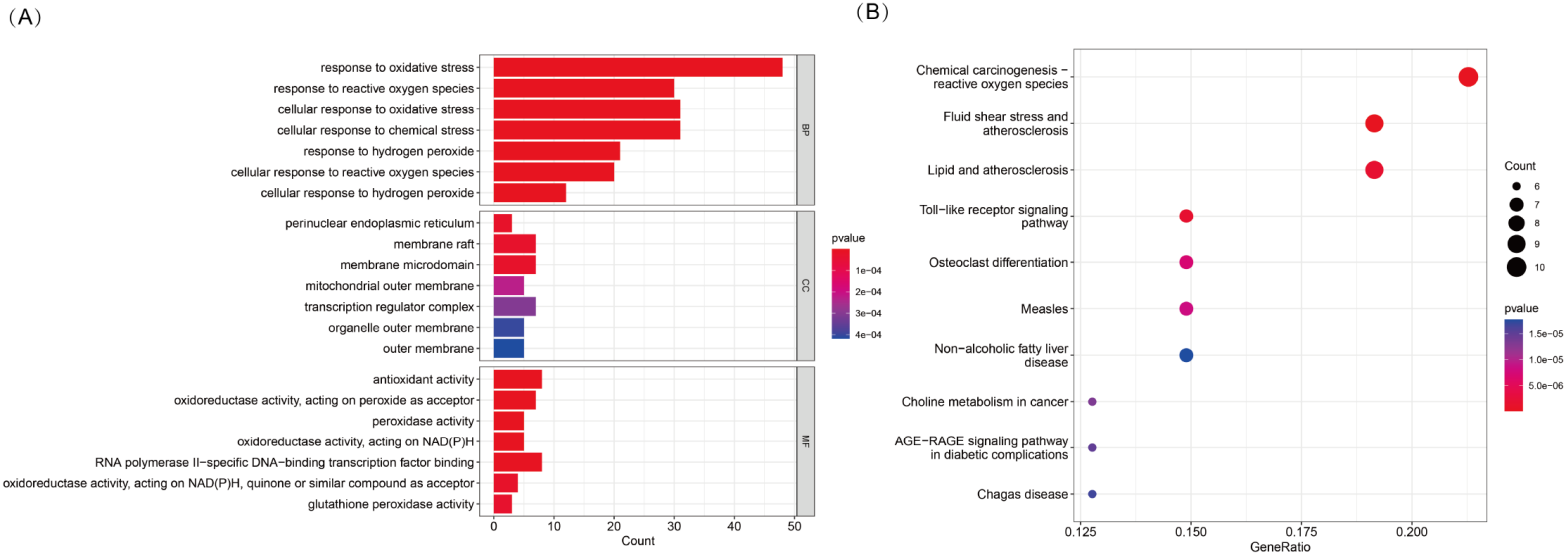
**(A)** KEGG pathway enrichment analysis of 53 DE-OSRGs, displayed as a bar plot. **(B)** GO enrichment analysis of 53 DE-OSRGs, visualized using a bubble plot, covering biological processes (BP), cellular components (CC), and molecular functions (MF).

To explore the potential biological functions and pathways associated with DE-OSRGs in TED, we conducted GO and KEGG pathway enrichment analyses. The GO enrichment analysis (**Figure 2A**) revealed the functional involvement of DE-OSRGs across biological processes (BP), cellular components (CC), and molecular functions (MF). In the BP category, genes were significantly enriched in cellular responses to oxidative stress, reactive oxygen species, and hydrogen peroxide, reinforcing the central role of oxidative stress in TED pathophysiology. In the CC category, enrichment in mitochondrial outer membrane, organelle outer membrane, and membrane microdomains highlights the critical role of mitochondria in TED progression and its potential association with oxidative damage. In the MF category, genes were significantly associated with antioxidant activity, peroxidase activity, and oxidoreductase activity (acting on NAD(P)H, quinones, or peroxides), suggesting that oxidative stress regulation and free radical scavenging mechanisms may be pivotal in TED development.

In the KEGG pathway analysis (**Figure 2B**), DE-OSRGs were significantly enriched in pathways such as chemical carcinogenesis—ROS, Toll-like receptor signaling, and the AGE-RAGE signaling pathway. These findings indicate a strong association between oxidative stress and inflammatory responses, metabolic dysregulation, and disease progression in TED. Additionally, the enrichment of pathways related to fluid shear stress and atherosclerosis as well as lipid metabolism and atherosclerosis suggests that vascular dysfunction and oxidative stress-induced endothelial damage may play a crucial role in TED pathogenesis.

### 3.4 Identification of five diagnostic OS-DEGs for TED

**Figure 3:**
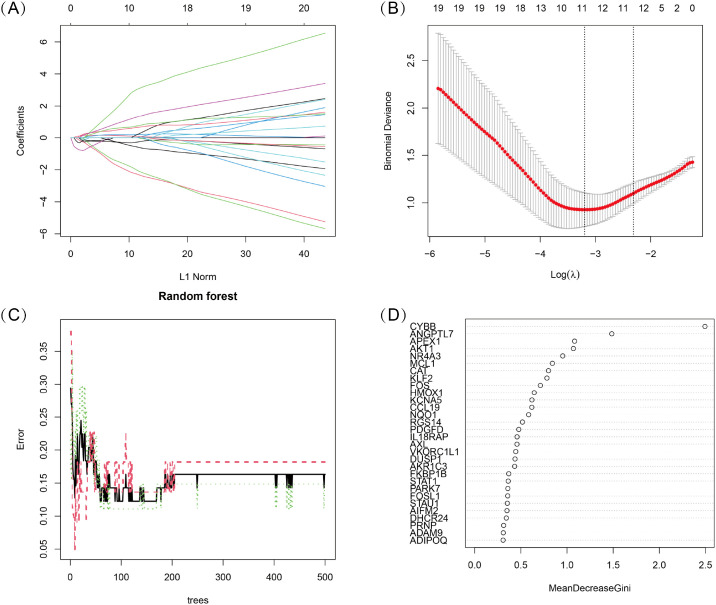
**(A, B)** LASSO logistic regression with 10-fold cross-validation was used to optimize the penalized parameter and identify key oxidative stress-related diagnostic genes for TED. **(C, D)** Random Forest (RF) algorithm was applied to evaluate the importance of oxidative stress-related candidate genes. Genes were ranked based on their Mean Decrease Gini Index, and the model’s error rate was analyzed.

**Figure 4:**
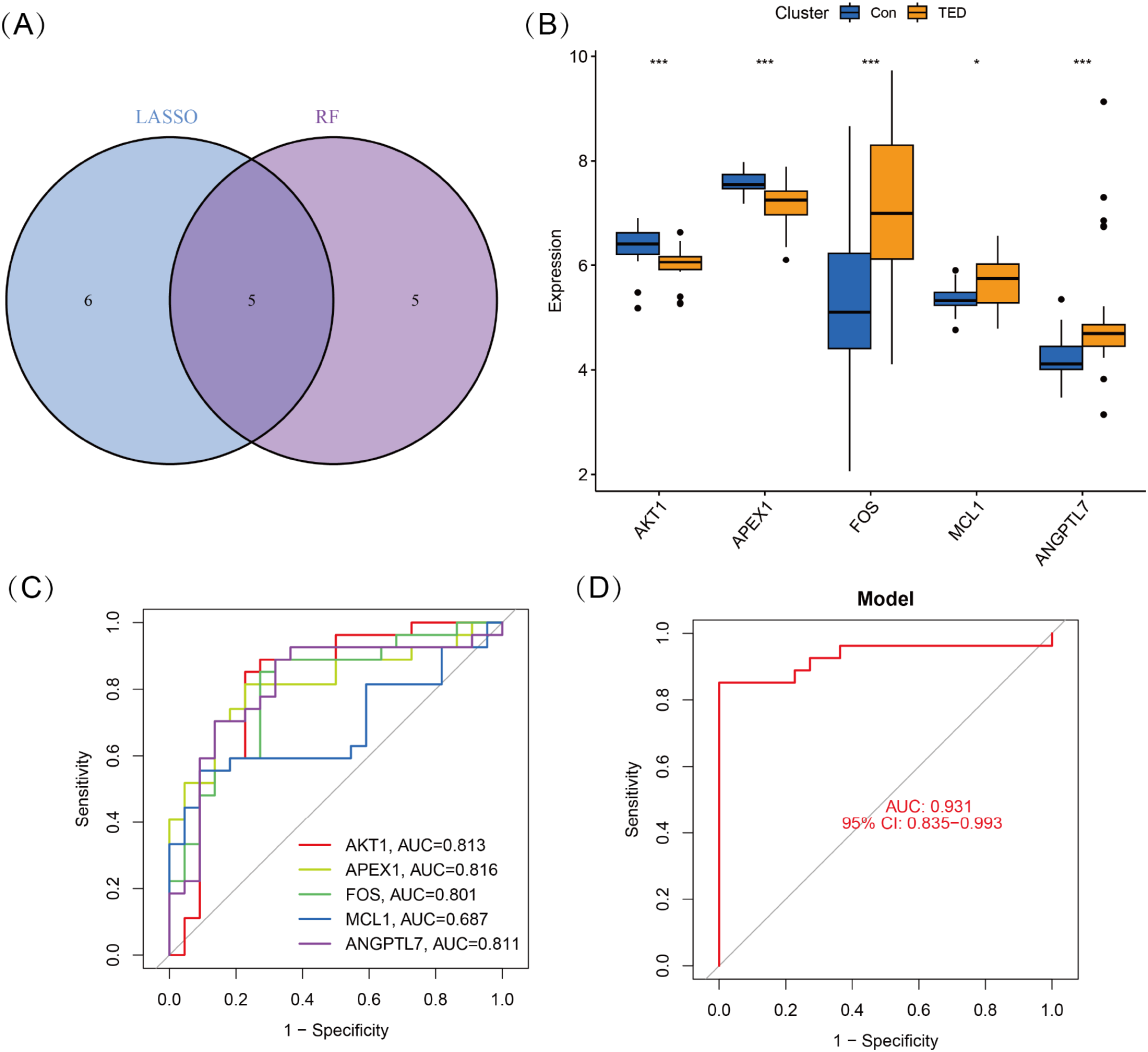
**(A)** Five DSGs were identified as oxidative stress-related diagnostic genes through the intersection of genes selected by LASSO and RF algorithms. **(B)** Boxplots displaying the expression levels of the five identified DSGs in TED and control groups. The expression differences between the two groups were statistically significant (*p* < 0.05), indicating their potential role in TED pathogenesis. **(C)** Receiver operating characteristic (ROC) curves for each DSG, illustrating their diagnostic performance. **(D)** The combined diagnostic model incorporating all five DSGs achieved an AUC of 0.931 (95% CI: 0.835–0.993), demonstrating its high predictive accuracy for distinguishing TED from control samples. Statistical significance: *P < 0.05; ***P < 0.001.

To identify key oxidative stress-related differentially expressed genes (OS-DEGs) for TED, we applied LASSO regression and random forest (RF) algorithms to screen significant genes that could effectively distinguish TED patients from healthy individuals (**Figures 3A–D**). The genes identified by these two machine learning methods were then intersected, yielding five diagnostic signature genes (DSGs), including *AKT1, APEX1, FOS, MCL1*, and *ANGPTL7* for further analysis (**Figures 4A, B**). To assess the ability of these individual genes to differentiate TED from normal samples, ROC curves were constructed. The AUC values for each gene were as follows: *AKT1* (AUC = 0.813), *APEX1* (AUC = 0.816), *FOS* (AUC = 0.801), *MCL1* (AUC = 0.687), and *ANGPTL7* (AUC = 0.811), demonstrating their potential as diagnostic biomarkers for TED (**Figure 4C**). Furthermore, a diagnostic model incorporating these five DSGs was constructed and evaluated using ROC analysis, achieving an AUC of 0.931 (95% CI: 0.835–0.993), indicating excellent diagnostic performance (**Figure 4D**). These findings suggest that these five oxidative stress-related genes could serve as potential biomarkers for TED diagnosis and provide insights into the role of oxidative stress in TED pathogenesis.

### 3.5 Construction of the nomogram model

**Figure 5:**
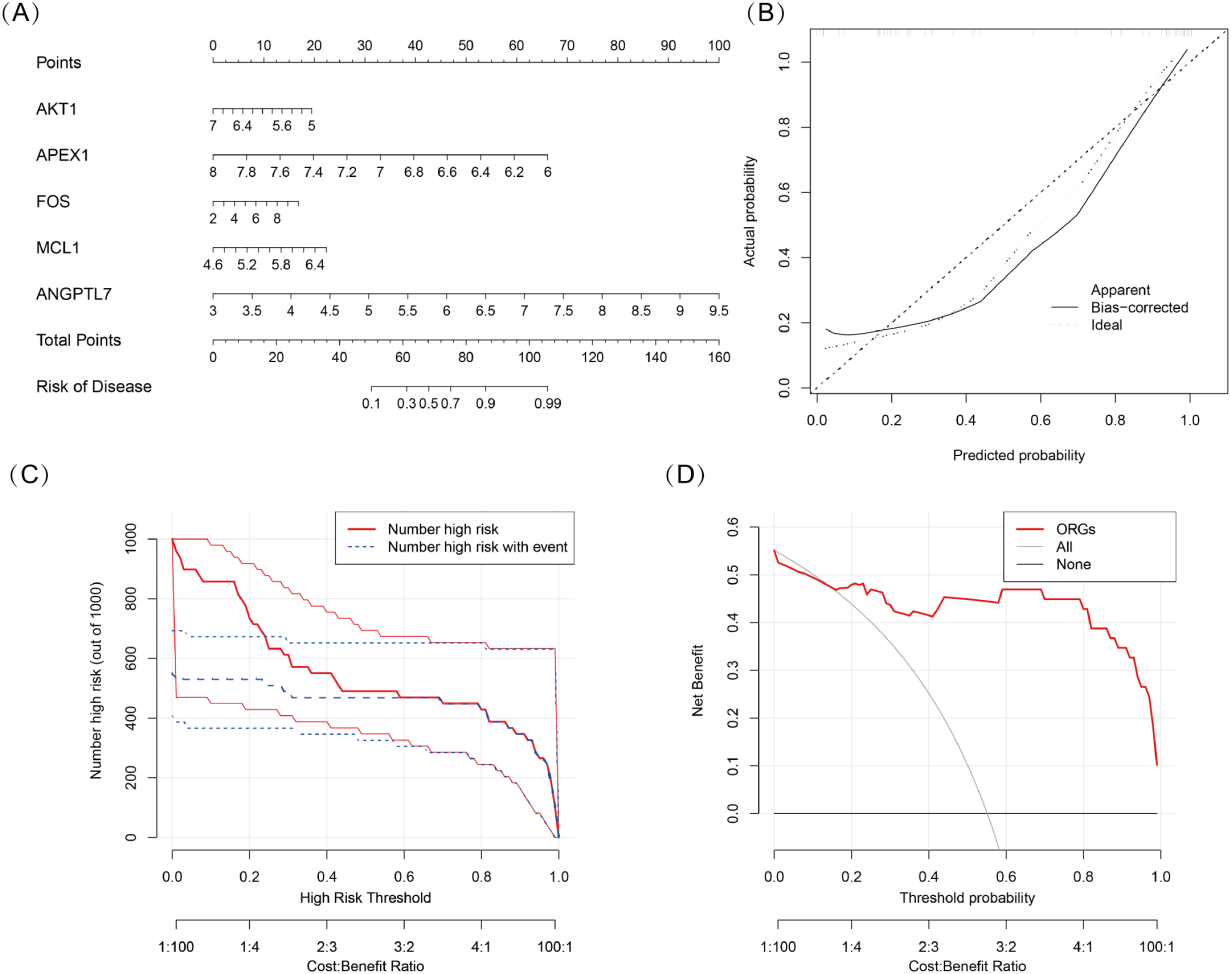
**(A)** Nomogram model based on five oxidative stress-related DSGs (AKT1, APEX1, FOS, MCL1, and ANGPTL7) for predicting the occurrence of TED. Each gene is assigned a score, and the total score corresponds to the predicted TED risk. **(B)** Calibration curve assessing the predictive accuracy of the nomogram model. The curve shows a close alignment between the predicted and actual TED risk, indicating good model reliability. **(C)** Clinical impact curve evaluating the potential impact of the model in a clinical setting. The “number high risk” curve closely follows the “number high risk with event” curve, suggesting that the model effectively identifies high-risk TED patients. **(D)** Decision curve analysis (DCA) demonstrating the clinical utility of the nomogram model. The net benefit curves suggest that the model may be useful for guiding clinical decision-making.

We developed a nomogram model for five DSGs using logistic regression (**Figure 5A**). The predictive performance of the model was evaluated using calibration curves, which demonstrated a small deviation between the predicted and actual risk of TED (**Figure 5B**). As shown in **Figure 5C**, the results suggest that the nomogram model may assist in clinical decision-making by providing an individualized risk assessment for TED patients. Additionally, a clinical impact curve (**Figure 5D**) was generated based on DCA. The “number high risk” curve, representing individuals classified as high risk, closely aligns with the “number high risk with event” curve, indicating that the nomogram model has reasonable predictive accuracy and may be useful for risk stratification in TED.

### 3.6 Single-gene GSEA analysis of *MCL1*, *FOS*, and *ANGPTL7*

**Figure 6:**
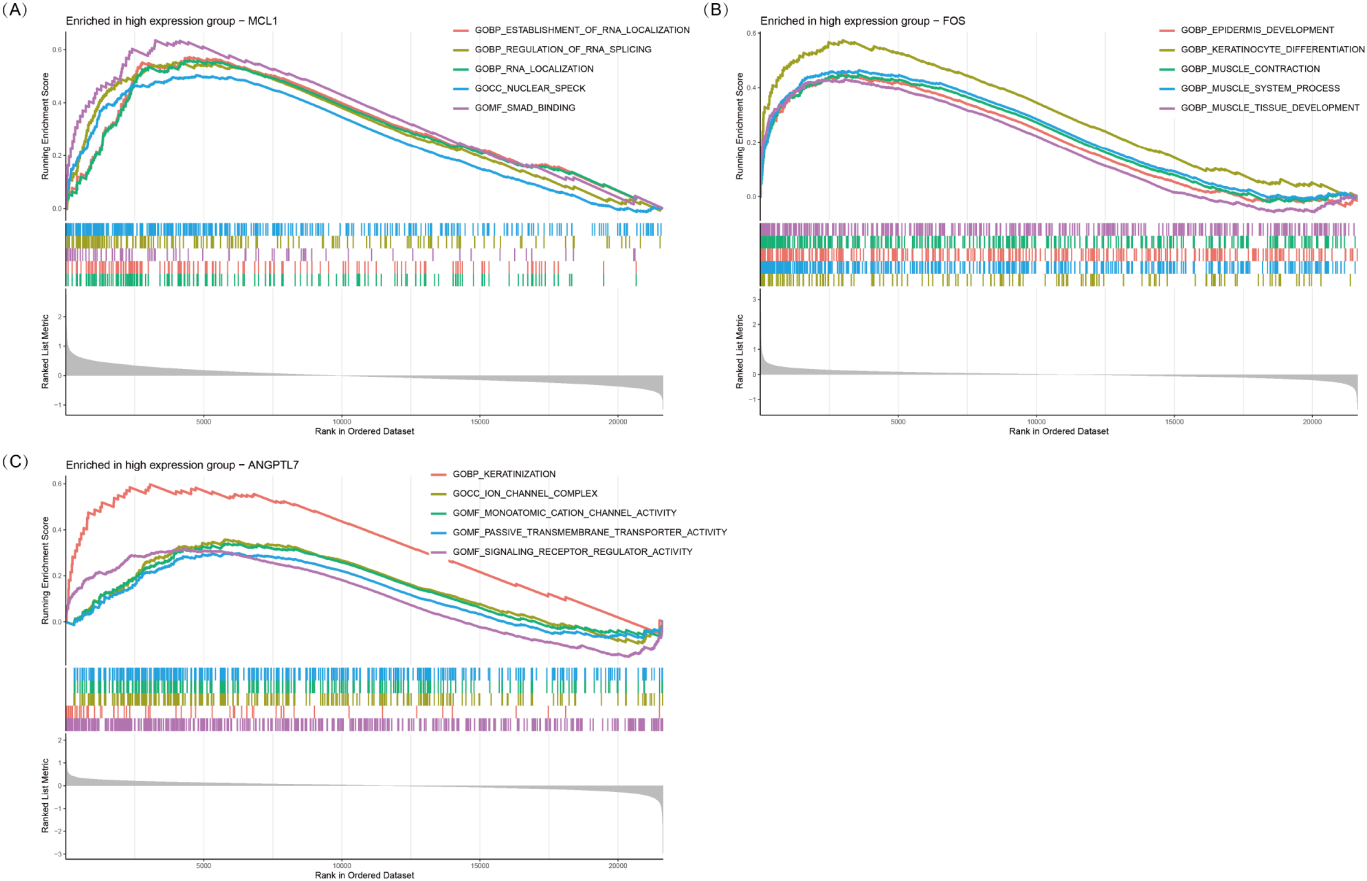
GSEA plots of GO terms enriched in the high-expression groups of *MCL1* **(A)**, *FOS* **(B)**, and *ANGPTL7* **(C)**. The top five significantly enriched GO pathways (*p* < 0.05) associated with high expression of each gene are shown.

Single-gene GSEA analysis revealed that three oxidative stress-related genes, *viz.*, *MCL1, FOS*, and *ANGPTL7*, were significantly associated with multiple biological processes in TED (**Figures 6A–C**). *MCL1* was primarily enriched in RNA metabolism and regulatory processes, including establishment of RNA localization (GOBP_ESTABLISHMENT_OF_RNA_LOCALIZATION), regulation of RNA splicing (GOBP_REGULATION_OF_RNA_SPLICING), and RNA localization (GOBP_RNA_LOCALIZATION). Additionally, it was enriched in nuclear speck formation (GOCC_NUCLEAR_SPECK) and SMAD binding (GOMF_SMAD_BINDING), suggesting that *MCL1* plays a crucial role in RNA metabolism and gene expression regulation and may influence TED pathogenesis via the TGF-β/SMAD-mediated inflammation and fibrosis pathway.

*FOS* was significantly enriched in epithelial development and muscle function-related processes, including epidermis development (GOBP_EPIDERMIS_DEVELOPMENT), keratinocyte differentiation (GOBP_KERATINOCYTE_DIFFERENTIATION), muscle contraction (GOBP_MUSCLE_CONTRACTION), and muscle tissue development (GOBP_MUSCLE_TISSUE_DEVELOPMENT). These results suggest that *FOS* may play a pivotal role in eyelid barrier repair, fibroblast activation, and extraocular muscle remodeling, potentially contributing to extraocular muscle hypertrophy and motility dysfunction in TED.

*ANGPTL7* was predominantly enriched in keratinization, ion channel function, and signaling receptor regulation-related processes, including keratinization (GOBP_KERATINIZATION), ion channel complex formation (GOCC_ION_CHANNEL_COMPLEX), and monatomic cation channel activity (GOMF_MONOATOMIC_CATION_CHANNEL_ACTIVITY). Additionally, it was associated with passive transmembrane transporter activity (GOMF_PASSIVE_TRANSMEMBRANE_TRANSPORTER_ACTIVITY) and signaling receptor regulator activity (GOMF_SIGNALING_RECEPTOR_REGULATOR_ACTIVITY). These findings indicate that *ANGPTL7* may be involved in orbital barrier function, intercellular communication, and inflammation signaling regulation, ultimately contributing to inflammation and fibrosis progression in TED.

### 3.7 FOS, MCL1, and ANGPTL7 are upregulated in orbital tissues of TED patients

**Figure 7:**
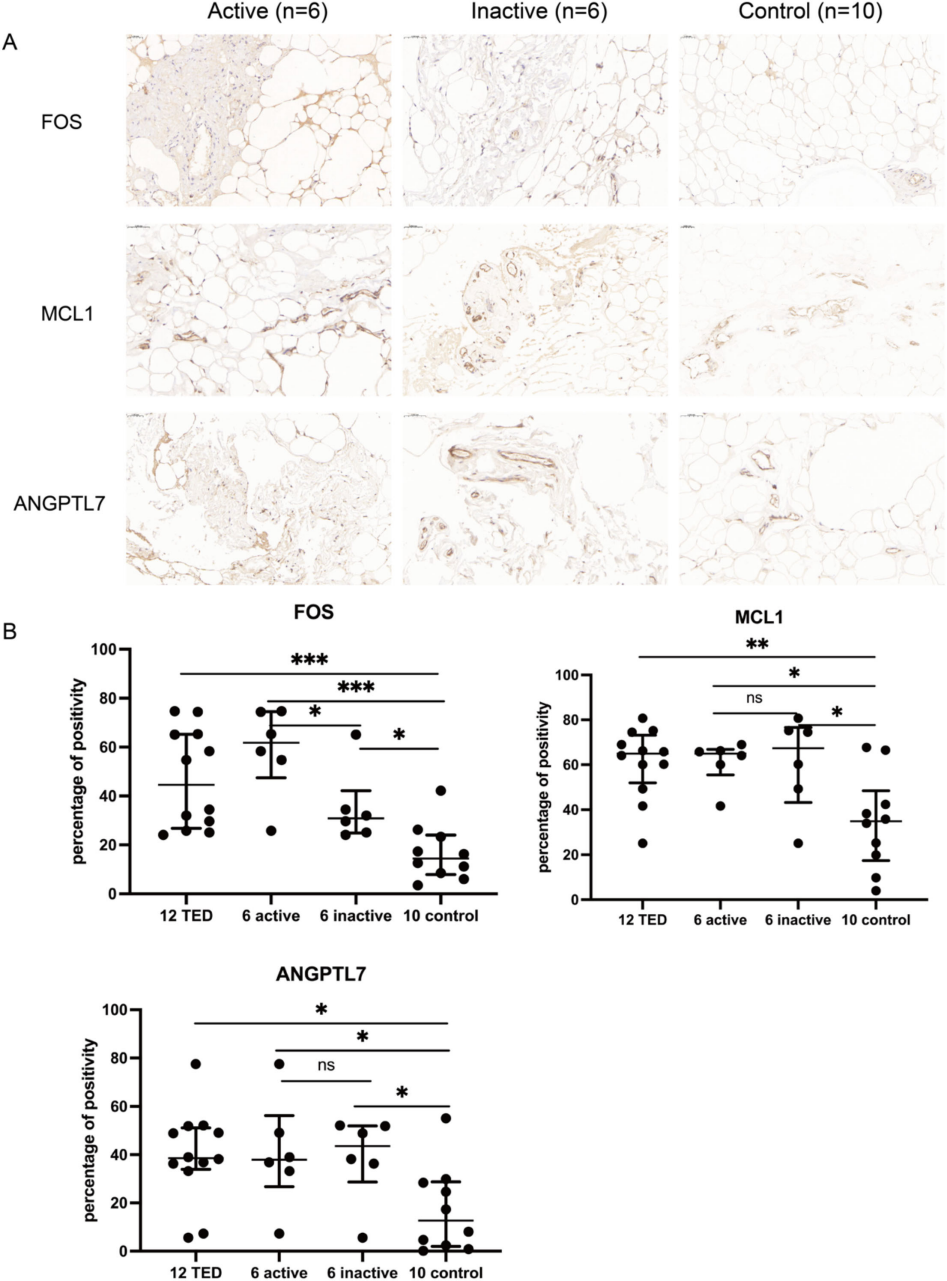
**(A)** Immunohistochemistry staining results of FOS, MCL1, and ANGPTL7 in the orbital connective/adipose tissue of patients with active (n=6) or inactive TED (n=6) and controls (n=10). Compared with normal controls, staining of FOS, MCL1, and ANGPTL7 was markedly increased in orbital tissues from TED patients. Notably, subgroup analysis showed significantly higher FOS expression in active TED compared to inactive TED, while no differential expression of MCL1 or ANGPTL7 was observed between the two TED subgroups. Bars represent 0.050 mm. **(B)** Percent positivity of FOS, MCL1, and ANGPTL7 in different orbital connective tissues from active TED (n=6), inactive TED (n=6), and control patients (n=10). Compared to controls, the percent positivity of FOS, MCL1, and ANGPTL7 was significantly higher in orbital connective tissues from patients with TED. FOS exhibited a high, moderate, and low percent positivity in the orbital tissues of active TED, inactive TED, and control groups, respectively. In contrast, MCL1 and ANGPTL7 showed similarly high expression in both active and inactive TED groups, with no significant difference between them, but both were significantly elevated compared to controls. *p < 0.05, **p < 0.01, ***p < 0.001; ns, not significant.

Quantitative immunohistochemical analysis revealed that the expression levels of FOS (P < 0.001), MCL1 (P = 0.003), and ANGPTL7 (P = 0.011) were significantly higher in the orbital connective tissues of TED patients compared to controls (**Figure 7**, **Table 2**). Further subgroup analysis showed that FOS expression was significantly elevated in active TED compared to inactive TED (*p* = 0.030), whereas MCL1 and ANGPTL7 levels were similarly increased in both TED subgroups without statistically significant difference. In contrast, significant differences were not observed in the staining of orbital adipose tissues (**Table 2**).

**Table 2:** Statistical analysis of immunohistochemical staining of orbital connective tissue and orbital adipose tissue collected from patients with TED and control subjects.

Molecules	TED (n=12)	Control (n=10)	*p1*	Active TED (n=6)	Inactive TED (n=6)	*p2*	*p3*	*p4*
Orbital connective tissue								
FOS	47.6 ± 21.0	16.7± 11.5	*<0.001*	60.1 ± 18.9	35.1 ± 15.2	*<0.001*	*0.030*	*0.016*
MCL1	61.0 ± 15.7	34.4 ± 21.2	*0.003*	61.2 ± 10.0	60.8 ± 21.0	*0.012*	*0.974*	*0.029*
ANGPTL7	39.7 ± 19.5	17.1 ± 17.6	*0.011*	40.5 ± 22.9	38.8 ± 17.6	*0.037*	*0.889*	*0.032*
Orbital adipose tissue								
FOS	22.4 ± 20.8	14.6 ± 15.3	*0.342*	29.1 ± 27.6	15.6 ± 9.0	*0.195*	*0.298*	*0.875*
MCL1	32.9 ± 10.6	21.4 ± 17.3	*0.071*	33.0 ± 14.9	32.7 ± 5.1	*0.193*	*0.962*	*0.079*
ANGPTL7	18.0 ± 10.7	12.2 ± 10.0	*0.201*	14.7 ± 8.4	21.3 ± 12.4	*0.606*	*0.306*	*0.126*

Data are presented as mean ± standard deviation. Significance values: *P1*, TED vs control; *P2*, active TED vs control; *P3*, active vs inactive TED; *P4*, inactive TED vs control.

## 4 Discussion

To the best of our knowledge, this study systematically explored for the first time the role of oxidative stress-related biomarkers in TED through transcriptomic sequencing, bioinformatics analysis, and immunohistochemical validation. The results highlight the significant role of oxidative stress-related molecules in the pathogenesis of TED and identify three key diagnostic oxidative stress-related genes (FOS, MCL1, and ANGPTL7) as potential biomarkers. These genes exhibited strong diagnostic capabilities, with a combined predictive model achieving an AUC of 0.931, indicating good discriminatory ability between TED patients and healthy controls. Notably, immunohistochemistry confirmed elevated expression levels of the three oxidative stress-related proteins (FOS, MCL1, and ANGPTL7) in orbital tissues from TED patients compared to healthy controls, suggesting their potential involvement in disease pathogenesis.

Further GO and KEGG enrichment analyses revealed potential molecular mechanisms underlying oxidative stress in the development of TED. GO analysis showed that DE-OSRGs were significantly enriched in cellular responses to oxidative stress, ROS metabolism, and mitochondrial dysfunction, emphasizing the role of mitochondrial oxidative damage in TED progression. KEGG analysis revealed that DE-OSRGs are involved in oxidative stress-related signaling pathways, including the Toll-like receptor pathway, AGE-RAGE signaling, and chemical carcinogenesis-ROS pathways, all of which are known to be associated with chronic inflammation and tissue remodeling. Additionally, the enrichment of DE-OSRGs in pathways related to vascular dysfunction and atherosclerosis suggests that endothelial oxidative stress may play a role in the orbital tissue remodeling and progression of TED.

FOS (c-Fos) is an immediate early gene that encodes the transcription factor c-Fos, which plays a crucial role in cell proliferation, differentiation, and survival. Fos can form an Activator Protein-1 (AP-1) complex with Jun family proteins to regulate the expression of specific genes (24). FOS is significantly upregulated in the extraocular muscles of TED patients, with RNA levels significantly higher than in healthy controls (22) and a transcriptomic and our bioinformatics analyses also revealed that FOS expression is elevated in TED (12, 23). In addition, the single-cell RNA sequencing study revealed increased expression of FOS in TED, primarily produced by lipofibroblasts with pro-adipogenic roles, which are involved in inflammation and oxidative stress (21). Our study further confirmed through immunohistochemistry that FOS protein expression is markedly elevated in TED patients. Furthermore, FOS was significantly upregulated in active TED tissues relative to inactive TED, with a statistically significant difference between the two groups.

In other disease models, FOS promotes oxidative stress, inflammation, and fibrosis through the AP-1 signaling pathway. For instance, in cardiac tissue, FOS induces ROS generation and myocardial fibrosis, and inhibiting FOS can significantly reduce inflammation (25). Furthermore, FOS is critically involved in Gli1 depletion-induced oxidative stress and apoptosis (26). Elevated expression of FOS aggravates oxidative damage and promotes cell death, thereby contributing to the progression of intervertebral disc degeneration. Notably, targeting FOS inhibition may serve as a potential therapeutic strategy to mitigate disc degeneration (26).

GSEA analysis indicates that FOS is primarily enriched in processes related to epithelial development and muscle function, suggesting that it may play a key role in eyelid barrier repair, fibroblast activation, and extraocular muscle remodeling, which are closely related to extraocular muscle hypertrophy and functional abnormalities in TED. A previous study demonstrated that members of the FOS family undergo transient upregulation in human skeletal muscle following exercise, indicating their pivotal role in modulating muscle-specific gene expression, promoting remodeling, and contributing to adaptation processes (27). Furthermore, reanalysis of the original data from our previous high-throughput proteomics study on TED indicated that FOS is engaged in numerous molecular pathways and participates in diverse biological processes (5). This further supports the idea that FOS might promote pathological remodeling of TED extraocular muscles through oxidative stress and inflammation regulation mechanisms.

MCL1 (Myeloid Cell Leukemia-1), a member of the BCL-2 family, primarily functions to inhibit apoptosis, maintain mitochondrial homeostasis, and regulate oxidative stress. It influences cell survival and adaptability by regulating ROS production, mitochondrial autophagy, and long-chain fatty acid oxidation (28–30). Multi-omics analyses, including single-cell RNA sequencing (21) and transcriptomic (20, 23), demonstrated upregulation of MCL1 in TED orbital tissues, a finding validated by immunohistochemistry. Importantly, the single-cell RNA sequencing study revealed increased expression of MCL1 in TED, primarily produced by lipofibroblasts and T cells, which may be involved in inflammation and oxidative stress (21).

MCL1 plays a critical role in maintaining mitochondrial function and energy metabolism balance (28–30). In the oxidative stress environment related to TED, MCL1 may influence OFs survival and adaptability by regulating mitochondrial function and ROS balance. Our GSEA analysis indicates that MCL1 is mainly enriched in RNA metabolism and SMAD signaling pathways, suggesting that it may impact TED’s pathological progression through TGF-β/SMAD-mediated inflammation and fibrosis. This finding aligns with previous studies, where TGF-β signaling promotes fibroblast activation and drives TED-related orbital fibrosis (1, 7, 9, 10). Alternative splicing of MCL1 has been shown in the literature to generate isoforms with distinct functions, influencing the balance between apoptosis and cell survival (31), which may be closely related to oxidative stress-associated splicing abnormalities in TED. Although there is currently no direct experimental evidence demonstrating its interaction with SMAD proteins, given its anti-apoptotic properties and the pivotal role of the TGF-β/SMAD signaling pathway in TED fibrosis, it is speculated that MCL1 may promote inflammation and fibrosis progression by affecting fibroblast survival or modulating TGF-β/SMAD-driven transcriptional programs. Therefore, MCL1 may play an important role in maintaining mitochondrial homeostasis, regulating ROS, inflammation and fibrosis signaling, and could be further investigated as a potential target for oxidative stress regulation in TED.

ANGPTL7 (Angiopoietin-like 7) is a member of the angiopoietin-like protein family and plays a key role in angiogenesis, metabolic regulation, and inflammation (32). ANGPTL7 is highly expressed in ocular tissues (such as the trabecular meshwork), and is primarily involved in intraocular pressure regulation, extracellular matrix remodeling, and is closely associated with glaucoma, inflammation, and oxidative stress (33–35). ANGPTL7 is significantly upregulated in TNF-α-induced human umbilical vein endothelial cells and mediates TNF-α-induced oxidative stress, with its inhibition reducing ROS levels, increasing NO and eNOS expression, inhibiting NF-κB activation, and enhancing the Nrf-2/HO-1 antioxidant pathway, thereby alleviating oxidative stress damage (34). Additionally, in a glaucoma model, ANGPTL7 promotes oxidative stress and mitochondrial dysfunction, while inhibiting its expression can alleviate oxidative damage (35).

Analysis of original transcriptomic and bioinformatics data demonstrates a significant upregulation of ANGPTL7 in the orbital tissue and or orbital fibroblasts of TED patients (19, 23). Consistently, our immunohistochemical investigation confirmed that ANGPTL7 protein expression is markedly increased in TED orbital connective tissues. The GSEA results show that ANGPTL7 is mainly enriched in processes related to keratinization, ion transport, and receptor signaling regulation, suggesting its potential role in orbital barrier function, inflammation modulation, and intercellular communication, which ultimately influences the inflammation and fibrosis progression in TED. ANGPTL7 has been reported to be associated with keratinization, as evidenced by its expression in corneal tissues and its role in preserving ocular surface integrity (36). Therefore, ANGPTL7 may play a key role in the orbital tissue remodeling and oxidative stress process related to TED and warrants further investigation into its specific regulatory mechanisms and potential therapeutic value.

In addition, although no direct interactions among FOS, MCL1, and ANGPTL7 have been reported in the current literature, all three molecules are critically involved in the regulation of oxidative stress. FOS promotes oxidative stress and induces ROS generation through the AP-1 signaling pathway (24–26), MCL1 regulates mitochondrial function and ROS balance (28–30), and ANGPTL7 promotes oxidative stress, mitochondrial dysfunction, and increased ROS levels (32–35). Given their respective roles in ROS production and mitochondrial dysfunction, it is plausible that these molecules may functionally interact in the oxidative stress-driven pathogenesis of TED. Further mechanistic studies are needed to elucidate their potential crosstalk.

The clinical significance and potential therapeutic value of these findings are noteworthy. Previous studies have demonstrated the pivotal role of oxidative stress in TED, leading to the consideration of antioxidant therapies as potential interventions (12, 13, 15, 16). For example, selenium supplementation has been shown to alleviate TED symptoms and reduce oxidative damage (1, 2, 13). The three diagnostic oxidative stress-related proteins identified in this study could serve as potential targets for precision treatment. Furthermore, the nomogram model developed in this study shows strong predictive capability in clinical assessments, suggesting that integrating these biomarkers into clinical detection could aid in early screening and risk stratification, and dynamic monitoring of treatment responses in TED patients. For the secreted protein ANGPTL7, its level in peripheral blood or tear fluid can be investigated to evaluate potential correlations with inflammation or disease severity in TED. In contrast, for the nuclear protein FOS and the intracellular protein MCL1, their roles in the oxidative processes of TED can be elucidated through further cellular experiments.

However, this study has several limitations. Firstly, the analysis is based on transcriptomic data, which does not comprehensively capture the effects of epigenetic modifications and post-transcriptional regulation on oxidative stress mechanisms in TED. As miRNA-mediated post-transcriptional regulation, DNA methylation, and histone modifications play important roles in inflammatory and autoimmune diseases, future studies could further explore how these mechanisms influence the expression and function of oxidative stress-related biomarkers in TED. Secondly, although immunohistochemical validation was performed, further functional studies (e.g., gene knockdown or overexpression in orbital fibroblasts) are required to clarify their specific roles in the oxidative stress–related progression of TED. Additionally, the dataset used in this study was derived from a relatively small cohort, and future studies with larger, multi-center cohorts are required to validate the reliability of these findings and further assess their clinical applicability.

In conclusion, this study identified three oxidative stress-related proteins (FOS, MCL1, and ANGPTL7) as potential biomarkers for TED and explored their roles in its pathogenesis. Bioinformatics analysis and immunohistochemical validation demonstrated involvement of these genes in oxidative stress pathways and may contribute to TED progression. The nomogram model demonstrated strong diagnostic potential, suggesting its value for early screening and risk assessment in TED. Further research is needed to validate these findings and assess their clinical applicability.

## Data Availability

Publicly available datasets were analyzed in this study. This data can be found here: http://www.ncbi.nlm.nih.gov/geo/, accession number GSE58331.
