# Supplementary figures and images for: Oxidative stress-related biomarkers in thyroid eye disease: evidence from bioinformatics analysis and experimental validation
Source: Front Immunol. 2025 Aug 18;16:1635712. doi: 10.3389/fimmu.2025.1635712 (PMC12400331; doi:10.3389/fimmu.2025.1635712)

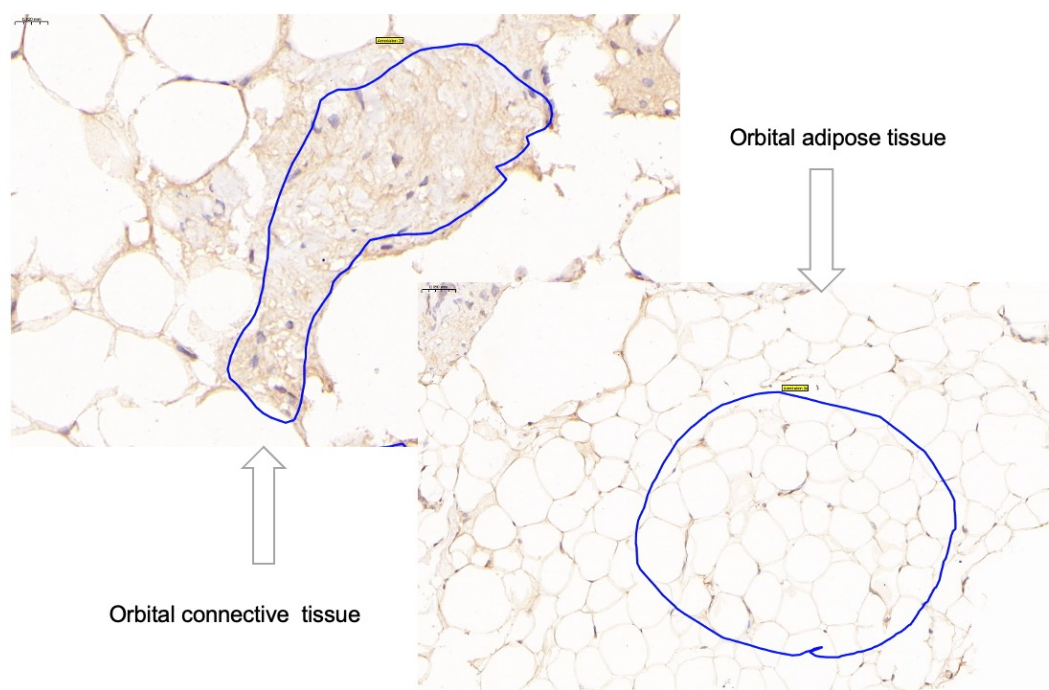

**Supplementary Figure S1**

Supplement: Supplementary file 1 [file DataSheet1.pdf]
